# Nanocrystalline Electrodeposited Fe-W/Al_2_O_3_ Composites: Effect of Alumina Sub-microparticles on the Mechanical, Tribological, and Corrosion Properties

**DOI:** 10.3389/fchem.2019.00241

**Published:** 2019-04-16

**Authors:** Aliona Nicolenco, Antonio Mulone, Naroa Imaz, Natalia Tsyntsaru, Jordi Sort, Eva Pellicer, Uta Klement, Henrikas Cesiulis, Eva García-Lecina

**Affiliations:** ^1^Department of Physical Chemistry, Vilnius University, Vilnius, Lithuania; ^2^Institute of Applied Physics, Chisinau, Moldova; ^3^Department of Industrial and Materials Science, Chalmers University of Technology, Gothenburg, Sweden; ^4^CIDETEC, San Sebastián, Spain; ^5^Departament de Física, Universitat Autònoma de Barcelona, Bellaterra, Spain; ^6^Institució Catalana de Recerca i Estudis Avançats, Barcelona, Spain

**Keywords:** iron alloys, alumina, composite coatings, columnar growth, wear resistance

## Abstract

In this study, nanocrystalline Fe-W alloy and Fe-W/Al_2_O_3_ composite coatings with various contents of sub-microsized alumina particles have been obtained by electrodeposition from an environmentally friendly Fe(III)-based electrolyte with the aim to produce a novel corrosion and wear resistant material. The increase in volume fraction of Al_2_O_3_ in deposits from 2 to 12% leads to the grain refinement effect, so that the structure of the coatings change from nanocrystalline to amorphous-like with grain sizes below 20 nm. Nevertheless, the addition of particles to the Fe-W matrix does not prevent the development of a columnar structure revealed for all the types of studied coatings. The observed reduction in both hardness and elastic modulus of the Fe-W/Al_2_O_3_ composites is attributed to the apparent grain size refinement/amorphization and the nanoporosity surrounding the embedded Al_2_O_3_ particles. In the presence of 12 vol% of Al_2_O_3_ in deposits, the wear rate decreases by a factor of 10 as compared to Fe-W alloy tested under dry friction conditions due to the lowering of tribo-oxidation. The addition of alumina particles slightly increases the corrosion resistance of the coatings; however, the corrosion in neutral chloride solution occurs through the preferential dissolution of Fe from the matrix. The obtained results provide a possibility to integrate the nanocrystalline Fe-W/Al_2_O_3_ composite coatings into various systems working under dry friction conditions, for example, in high-temperature vacuum systems.

## Introduction

Nanocrystalline Fe-W coatings with tunable functional properties can be electrodeposited from environmentally friendly Fe(III)-based solution with a high current efficiency (up to 60–70%) (Nicolenco et al., 2017, 2018a). The W content in the alloys can be varied from a few at.% up to 25 at.% by fine control of experimental parameters. Previous studies (Nicolenco et al., 2018a) have shown that alloys with low W content have a soft magnetic character and relatively high saturation magnetization, making such alloys suitable for the design and fabrication of MEMS, for example, read-write heads or actuators. On the other hand, Fe-W alloy coatings with high W content (>20 at.%) are characterized by outstanding thermal stability (up to 600°C), good mechanical properties in a wide range of temperatures (10 ÷ 16 GPa) (Mulone et al., 2018a), and mirror-like smooth surface (Ra <100 nm) (Bobanova et al., 2009; Mulone et al., 2018b; Nicolenco et al., 2018a). Nevertheless, one of the drawbacks of Fe-W coatings is their rather low wear and corrosion resistance that remains an important issue for the applications of these nanoengineered materials as protective coatings and smart interfaces.

Fabrication of composite materials is one of the common approaches, which enables to combine the useful properties of the second-phase particles with those of the metallic matrix, thus rendering a novel material with the tailored characteristics. For example, the co-deposition of transition metal sulfides (MoS_2_ and WS_2_) in Ni-W and Ni-P coatings significantly reduced the friction coefficient of the composites due to the specific layered structure and high elasticity of the particles, which provide a self-lubricating effect (Cardinal et al., 2009; García-Lecina et al., 2013; He Y. et al., 2016). Many studies have been conducted on the electrodeposition of metallic matrix composites with oxide and carbide particles. Here, an improvement of the strength and corrosion resistance of the resulting composite coatings was obtained thanks to the high hardness, the oxidation and thermal resistance of the co-deposited particles (Hosseini et al., 2014; Anwar and Mulyadi, 2015; Bhogal et al., 2015; Bajwa et al., 2016). However, the presence of second phase particles in the coating is not a guarantee that the desired performance will be attained. Often the incorporation of particles causes porosity, bad adhesion and weak interface bonding with the matrix. All these effects can eventually reduce the overall performance of the surface, particularly, corrosion resistance (Starosta and Zielinski, 2004; Hu et al., 2009; Bajwa et al., 2016). Therefore, the appropriate design of the composite system is essential.

Composite coatings with alumina particles have been the most extensively studied among particles-reinforced composites. In addition of its cost effectiveness, alumina has high chemical stability and sufficiently high affinity to adsorb iron group metal ions on its surface, thus facilitating the co-deposition of composites with Co, Ni and their alloys (Wu et al., 2004; Man et al., 2014). Well-dispersed composites with Al_2_O_3_ particles typically show good corrosion resistance in the sulfate and chloride medium due to the partial blocking of corrosion pits by inert alumina nano- or microsized particles (Bajwa et al., 2016). The addition of alumina particles can also contribute to the increased hardness and strength of the coatings, as the particles restrain the grain growth and provide a dispersion strengthening effect described by Orowan mechanism (Beltowska-Lehman et al., 2012; Man et al., 2014;Bajwa et al., 2016; Wasekar et al., 2016).

Several works exist in the literature correlating the mechanical characteristics of composite coatings with their tribological properties, giving relationships between the plasticity index (or hardness) and wear (Rupert and Schuh, 2010; García-Lecina et al., 2013; Bajwa et al., 2016). The studies performed mainly on Ni-based coatings showed that nanocrystalline materials with a higher ratio between hardness and elastic modulus, so-called elastic strain to failure, should better resist the plastic deformation, hence, resulting in lower wear (Leyland and Matthews, 2000; Rupert and Schuh, 2010). Nevertheless, the wear mechanism of Fe-containing coatings was ascribed to the combination of abrasive and adhesive wear considering tribo-oxidation as the driving factor. The high wear rates of Fe-W coatings could only be overcome by applying lubrication conditions (Bobanova et al., 2009; Nicolenco et al., 2018b). The tribological study performed on Ni-Fe/Al_2_O_3_ composites under dry friction conditions has also shown the negative and prevalent effect of Fe content in matrix on the wear resistance of the coatings, while the content of alumina particles had a minor effect (Starosta and Zielinski, 2004).

The aim of our work is to electrodeposit nanocrystalline Fe-W/Al_2_O_3_ composite coatings with various contents of incorporated sub-microsized alumina particles to improve the wear and corrosion characteristics of the coatings and make them suitable for various applications where aggressive conditions can exist. The effect of alumina particles' co-deposition on the composition and structure of the coatings, and their mechanical, tribological, and corrosion behavior is investigated.

## Experimental Part

Fe-W/Al_2_O_3_ composite coatings were electrodeposited from a glycolate-citrate Fe(III)-based electrolyte which contained alumina particles in suspension. The base electrolyte was composed of 1 M glycolic acid, 0.3 M citric acid, 0.1 M Fe_2_(SO_4_)_3_, and 0.3 M Na_2_WO_4_. Bath pH was adjusted to 7.0 with NaOH at room temperature. Sub-microsized alumina particles (Alfa Aesar 42572) with the concentration of 25, 50, and 100 g L^−1^ were added to the base electrolyte and the suspensions were stirred at 300 rpm for 24 h prior to first electrodeposition in order to hydrate the particles. Before starting the electroplating, the electrolytes were placed in an ultrasonic bath for 10 min to prevent agglomeration.

Electrodeposition was performed in a typical three-electrode cell, where a brass plate with an area of 4 cm^2^ was used as a working electrode, Ag/AgCl/KCl_sat_ as a reference electrode and a platinized titanium mesh as a counter electrode. The substrates were degreased in a hot commercial alkaline cleaner and activated in H_2_SO_4_ solution, followed by Ni-seed layer electrodeposition at 30 mA cm^−2^ for 1 min from sulfate-chloride bath operated at 65°C. The anode was placed parallel to the cathode at a distance of 4.8 cm. The electrolyte volume was kept at 200 mL. The bath temperature was maintained at 65°C. Electrodeposition was carried out at a constant cathodic current density of 40 mA cm^−2^ during 1 h, at a constant stirring rare of 200 rpm.

The morphology of the coatings was investigated by a Carl Zeiss Ultra Plus field emission scanning electron microscope (Zeiss, Jena). The chemical composition was analyzed on cross-sectional area with the energy dispersive X-ray spectroscopy (EDS) analysis tool AMETEK EDAX attached to the microscope operated at 15 kV. The particles size distribution and the volume fraction of co-deposited Al_2_O_3_ particles in the deposits was determined by the image analysis software ImageJ. It was determined that the majority of the alumina particles have the mean diameter between 50 and 150 nm, although some of the particles were forming agglomerates with 200–250 nm (Figure S1), probably due to the high surface energy of the powder. The contact profilometer Surftest SJ-210 was used to determinate the roughness (*R*_a_) of the coatings. A contact needle tip was scanning the surfaces horizontally along the 1.5 mm length. The crystallographic structure and phase composition of the obtained coatings were studied by means of X-ray diffraction (XRD), using a Rigaku MiniFlex II diffractometer with Cu Kα radiation (λ = 1.54183 Å) operated at 30 kV and 30 mA.

The crystallographic orientation of the grains in the deposited coatings was examined by the Electron Back Scattered Diffraction technique (EBSD) in a Leo 1550 Gemini Scanning Electron Microscope (SEM). The EBSD data were acquired with a Nordlys II detector (Oxford Instruments) using a step size of 25 nm. Post-processing of the acquired EBSD data was done with the HKL Channel 5 software (Oxford Instruments): noise reduction was performed by removal of wild spikes and extrapolation of non-indexed points (5 nearest neighbors required). Metallographic preparation of the samples was performed by mechanical polishing with a 50 nm finishing using OP-S silica suspension as the last step.

The mechanical properties were evaluated by means of nanoindentation using Nanoindenter XP from MTS equipped with a Berkovich pyramidal-shaped diamond tip under load-control mode on the cross-section of the coatings, previously embedded in epoxy resin and polished to mirror-like appearance. The maximum applied load was 50 mN to ensure that the lateral size of the imprint remained small compared to the total film thickness but still embraced both the matrix and the particles in the case of composite coatings. Fifty indentations were placed in the middle of the cross-section area of the coatings in order to avoid the influence from the resin. The metallographic preparation of the samples was performed by mechanical polishing with a 1 μm finishing using a diamond suspension as the last step. Both hardness (H) and reduced elastic modulus (E_r_) were derived from the initial part of the unloading indentation segments using the method of Oliver and Phar (Oliver and Pharr, 2004). In order to characterize the elastic-plastic response of the coatings under external deformation, the plasticity index (U_p_/U_tot_) was calculated as a ratio between plastic and total (plastic + elastic) energy during nanoindentation. The plastic energy was derived as the area between loading and unloading curves, while the total energy was calculated as the area between the loading curve and the displacement axis. All the extracted parameters were statistically treated, and the average values are reported.

The investigation of tribological behavior of electrodeposited coatings was carried out under dry friction conditions using ball-on-disk configuration sliding tests (CSM, model THT). A corundum ball of 6 mm diameter was the counter-body that moved against rigidly fixed coated samples for 500 m with a rotation speed of 4 cm s^−1^ (rotation diameter 6 mm). The applied load was 2 N. All the tests were performed in ambient air at 20 ± 2°C and 55% relative humidity. After the sliding tests, the debris were removed from the surface with dry cold air jet, and the samples were ultrasonically cleaned in ethanol in order to remove the remaining adhered debris prior to measuring the wear track profiles. The specific wear rate was defined as wear volume per unit distance and unit load (Bajwa et al., 2016).

Corrosion resistance of the Fe-W and Fe-W/Al_2_O_3_ coatings was investigated by potentiodynamic polarization and electrochemical impedance spectroscopy (EIS) techniques under open circuit conditions. A 250 mL three-electrode Flat Cell Kit (Princeton Applied Research, Oak Ridge, TN, USA) with a platinized titanium mesh and Ag/AgCl/NaCl_(3M)_ electrodes as counter and reference electrodes, respectively, was used to conduct the experiments. The potential of the working electrode was measured against the reference electrode with a Luggin Haber capillary tip. The exposed area of the sample was 1 cm^2^. The tests were performed in 0.1 M NaCl solution at room temperature. The open circuit potential was recorded for 15 min followed by a linear potentiodynamic sweep from −1 to 0 V with a scan rate of 1 mV s^−1^. EIS measurements were conducted by applying sinusoidal voltage with 5 mV (vs. OCP) amplitude in the frequency range 10 kHz−0.01 Hz, and the fitting of data has been performed using Z-View software.

## Results and discussion

### Effect of Alumina Particles on the Composition, Morphology, and Structure of the Fe-W Composite Coatings

Considerable attention in composite coatings electrodeposition was directed primarily toward determination of optimum conditions for their production, i.e., temperature, pH, current density, rotation speed, etc. (Low et al., 2006). However, it is often difficult to generalize the findings due to the large difference in experimental conditions. Therefore, in order to investigate the effect of sub-micron alumina particles on the composition, morphology and structure of Fe-W composite coatings, the same parameters as for electrodeposition of compact Fe-W alloys with high W content and high current efficiency were used, i.e., the deposition conditions applied were 65°C, pH 7, 40 mA cm^−2^, and 200 rpm. The different content of alumina particles in the deposits was achieved by variation of the concentration of alumina particles in the bath.

**Figure 1:**
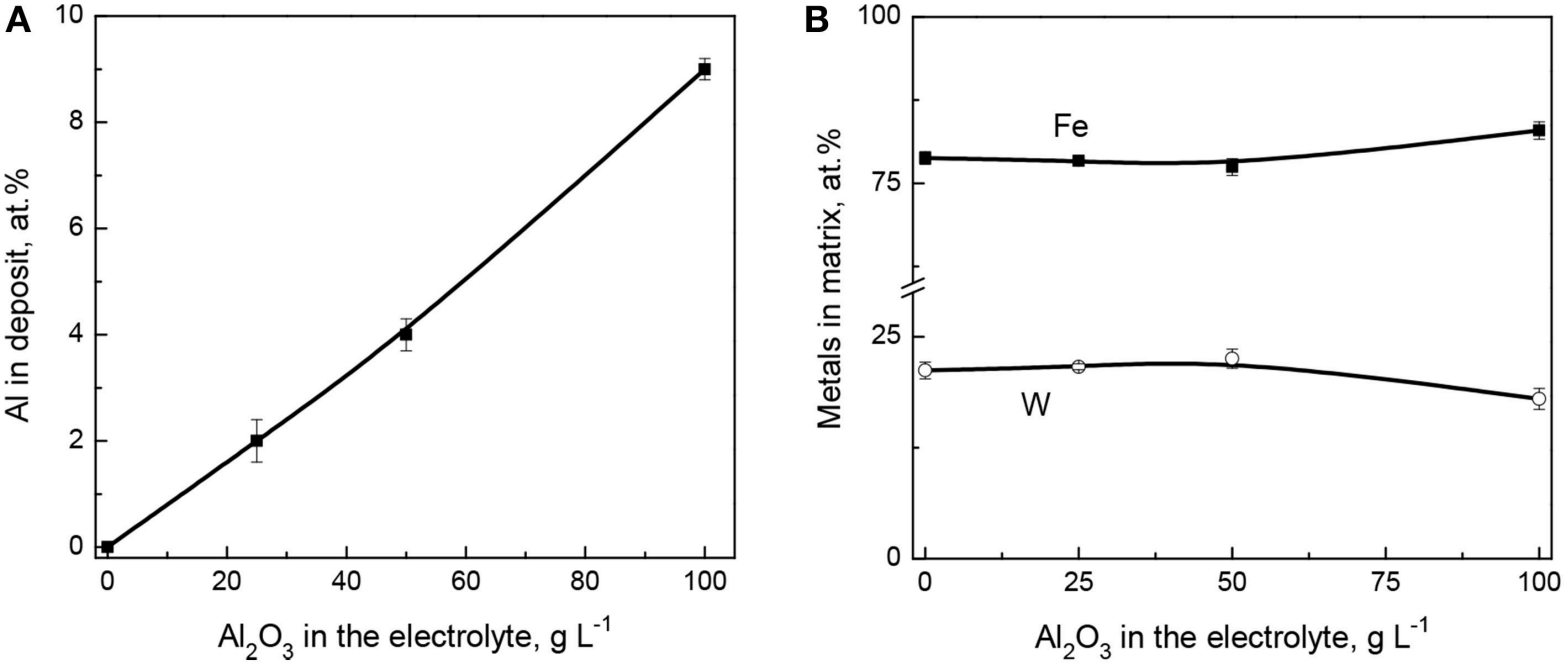
Effect of Al_2_O_3_ particles' concentration in the electrolyte on Al content in deposit **(A)** and on the composition of Fe-W matrix **(B)**. The coatings were obtained at 200 rpm, 40 mA cm^−2^, 65°C.

The compositional analysis of the electrodeposited coatings was performed on the cross-section area where two different regions were selected for examination: a wider area embracing Al_2_O_3_ particles, and a particle-free region (Fe-W matrix). Both regions were inspected at four different locations of each sample. The average atomic fraction of incorporated Al, and the Fe and W % contents in the matrix are shown in Figures 1A,B, respectively. It is seen that the content of aluminum (alumina particles) increases sharply with the increase of the particles concentration in electrolyte up to 100 g L^−1^. The maximum percentage of incorporated alumina is obtained at the maximum studied concentration of particles in solution, as predicted by Guglielmi's two-step adsorption model (Low et al., 2006; Wasekar et al., 2016).

In its simplest form, the mechanism of alumina particles co-deposition with Fe-W alloy can be described as follows: in a first step, suspended alumina particles are transported from the bulk of the solution to the cathode through the Nernst diffusion layer, where they become loosely adsorbed at the cathode's surface. It is supposed that the transportation of particles is not only due to convection force, but also due to the formation of ionic clouds (i.e., adsorption of charged metal complexes in the bulk electrolyte) on the particles surface, which move toward the cathode under electric field and thus drag the particles (Low et al., 2006; Wasekar et al., 2016). In a second step, the charge transfer reaction breaks the ionic shell surrounding the particles and thus they become irreversibly encapsulated into the growing metallic layer. Although the particles co-deposition mechanism has been studied by many authors and using different approaches, the Guglielmi's model still remains one of the most commonly adopted to describe the behavior of the particles during electrodeposition. It was actually validated for some binary alloys composites with inert particles, such as Ni-Fe/Al_2_O_3_ (Starosta and Zielinski, 2004; Torabinejad et al., 2016), Ni-W/SiC (Wasekar et al., 2016) and others (Low et al., 2006).

Taking into account the electrochemically inert nature of alumina particles one can expect that the composition of the metallic matrix should not be affected by the particles concentration in the solution unless their incorporation does not cause an apparent decrease of the active cathode surface area (Beltowska-Lehman et al., 2012). Indeed, as it is seen from Figure 1B, the incorporation of alumina particles does not influence on the W content in Fe-W matrix. However, slightly lower W content was achieved within the matrix when the concentration of particles in solution reached 100 g L^−1^. This is probably due to the partial blocking of the cathode area by non-conductive alumina, which resulted in an increased cathodic current density of the alloy deposition and a shift of cathodic polarization curve toward more negative potentials (Figure S2). As a result, deposition of coatings from the bath containing 100 g L^−1^ of Al_2_O_3_ occurs at slightly higher overpotential, that is −1.32 V compared to −1.25 V recorded in the case of Fe-W alloy deposition at the same cathodic current density applied (Figure S3). Thus, the W content in Fe-W matrix attains 21 at.% when the concentration of particles does not exceed 50 g L^−1^, and 17 at.% at higher alumina particles concentrations, respectively. Therefore, despite the non-conductive nature of Al_2_O_3_ particles, the current efficiency and the deposition rate remained rather unchanged as compared to Fe-W alloy deposition. These findings are consistent with other studies emphasizing the effect of alumina particles on the composition of binary alloy metallic matrix (Yari and Dehghanian, 2013; Wasekar et al., 2016).

**Figure 2:**
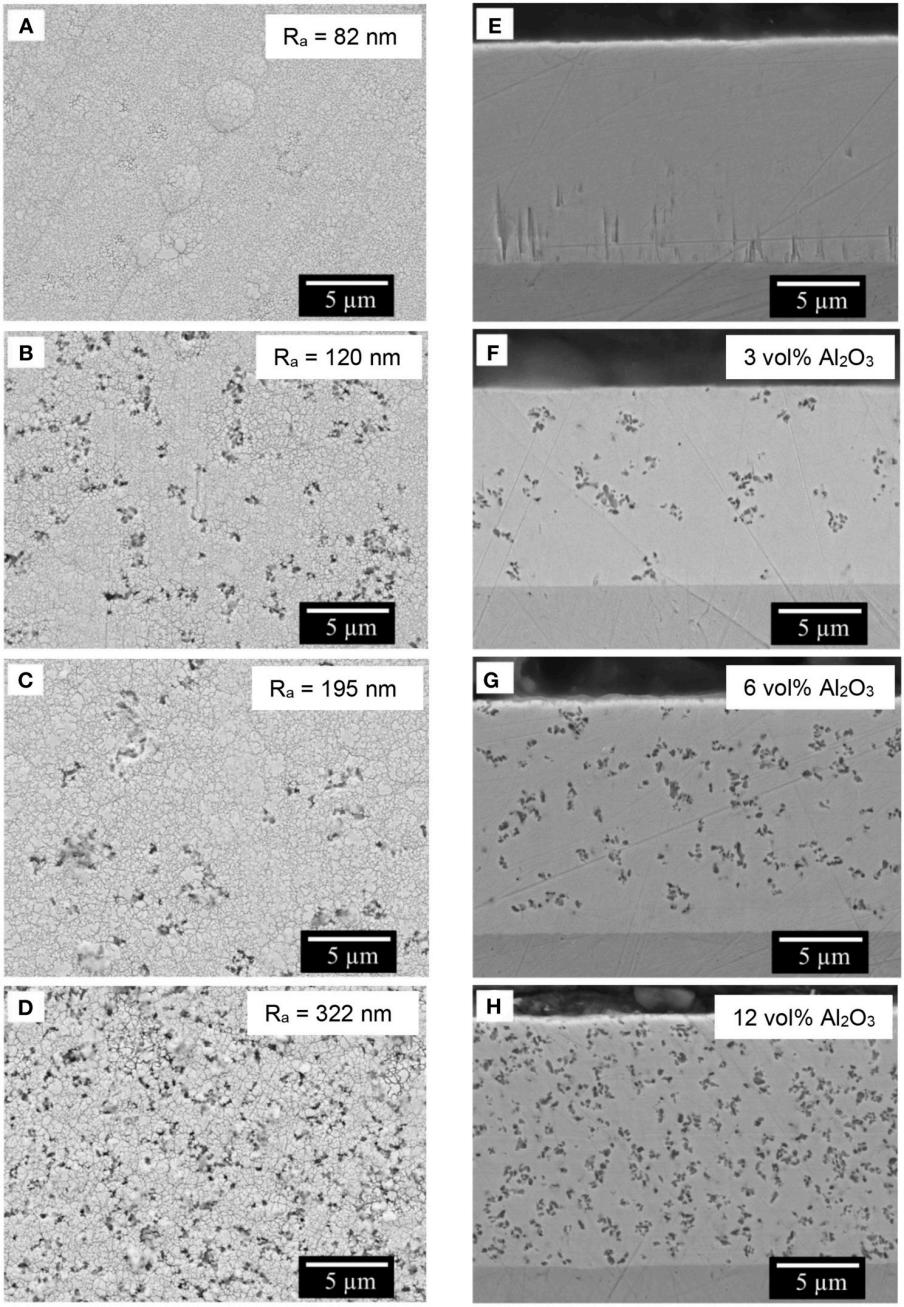
SEM images of the top surface **(A–D)** and the cross-section polished with 1 μm finishing **(E–H)** of the composite coatings obtained with different concentration of alumina in electrolyte: 0 g L^−1^ **(A,E)**, 25 g L^−1^ **(B,F)**, 50 g L^−1^ **(C,G)**, and 100 g L^−1^ **(D,H)**. The average roughness of the surface is indicated in the corresponding images **A–D**. In inserts **F–H** is the volume percentage of embedded alumina particles determined by corresponding image analysis. Electrodeposition was carried out at 40 mA cm^−2^, 65°C, 200 rpm.

The morphology of Fe-W alloy and Fe-W/Al_2_O_3_ composite coatings electrodeposited at the same conditions was analyzed by SEM and the representative images of the coatings surface are shown in Figures 2A–D (left). SEM images of Fe-W alloy surface depict a smooth, cracks-free globular structure with the average roughness of ~80 nm (Figure 2A). The addition of particles does not change the morphology of the coatings. However, the surface roughness increases gradually up to 300 nm due to protrusion of Al_2_O_3_ particles (Figures 2B–D). The panels e-g of Figure 2 (right) illustrate the corresponding cross-sectional images of the studied samples. Remarkably, the distribution of Al_2_O_3_ across the whole thickness of the coatings is uniform; although, the particles tend to incorporate in form of the small agglomerates with ~200 nm size. The volume percentage of embedded alumina particles was determined by the image analysis of the corresponding cross-sectional surface of the coatings. According to that, the volume fraction of the Al_2_O_3_ in deposit increases linearly from ~3 vol% to a maximum 12 vol% with the increase in particles concentration in the bath up to 100 g L^−1^. All the coatings have approximately the same thickness, about 14–15 μm, which confirms the minor effect of alumina particles on the deposition rate of the composites.

**Figure 3:**
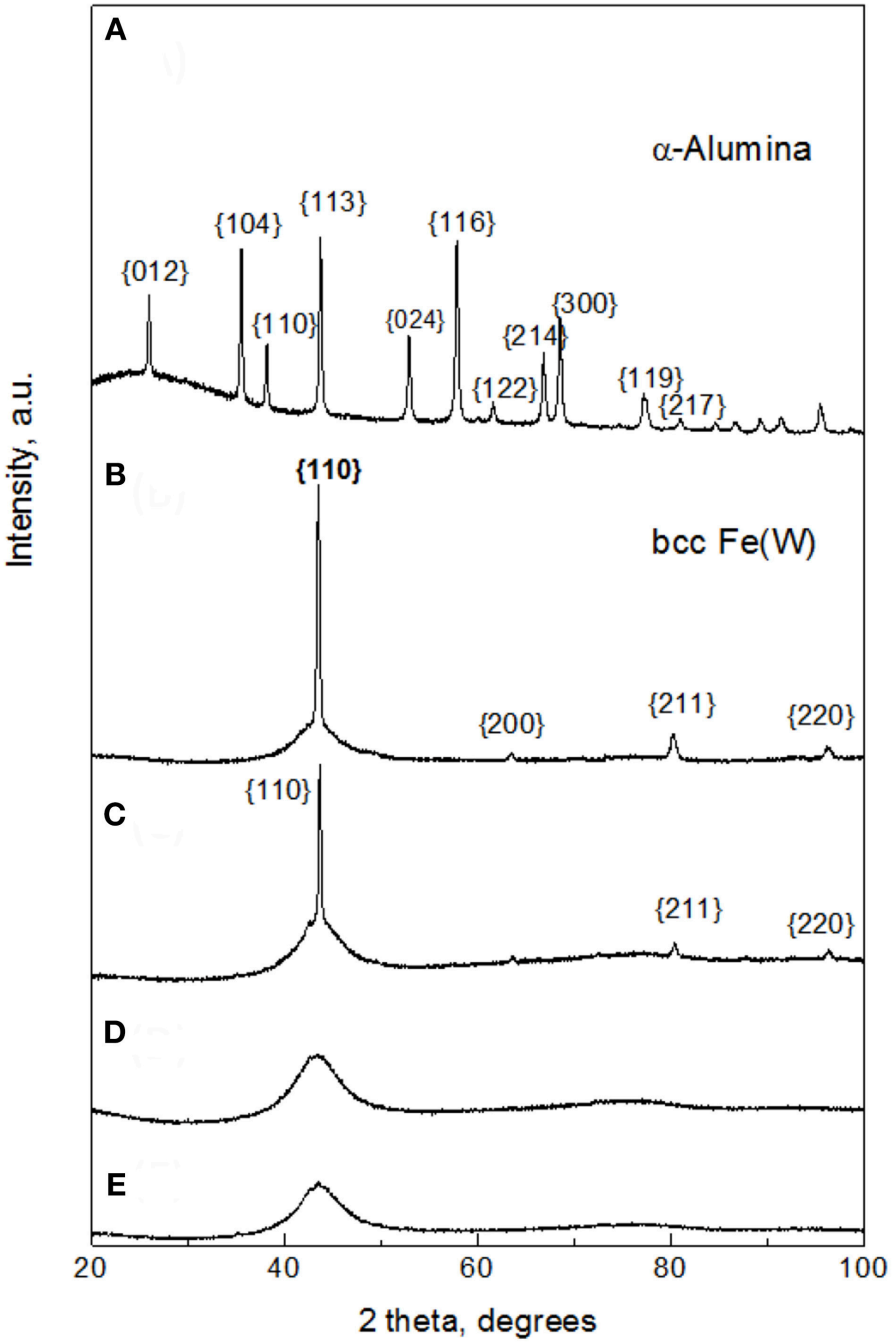
XRD patterns of sub-microsized alumina powder **(A)**; Fe-W coating **(B)**, and Fe-W/Al_2_O_3_ composite coatings obtained with different concentration of alumina in electrolyte: 25 g L^−1^ **(C)**, 50 g L^−1^ **(D)**, and 100 g L^−1^ **(E)**.

The microstructural changes caused by addition of Al_2_O_3_ particles to the Fe-W matrix were investigated by XRD and EBSD techniques. Figure 3A shows the XRD pattern of the sub-microsized alumina particles where the main peaks corresponding to rhombohedral Al_2_O_3_ were indexed. Remarkably, in the XRD patterns of Fe-W/ Al_2_O_3_ composites (Figures 3C,D) the peaks corresponding to the Al_2_O_3_ particles are not observed. It can be suggested that the amount of co-deposited alumina particles is insufficient to provide strong reflections. In fact, their amount ranges between 2 and 12 vol% (Figure 2) which roughly corresponds to <4 wt.%. This is, however, in the limit of X-Ray diffraction in a multi-component materials, where at least 2–3 wt.% is needed to have clear diffraction peaks (Newman et al., 2015).

The corresponding XRD pattern of the Fe-W alloy (Figure 3B) illustrates that the matrix of the composite coatings is composed of a crystalline body-centered cubic (bcc) single phase Fe(W) solid solution. In fact, the identified peaks are shifted to lower angles as compared to the characteristic peaks of bcc Fe due to the expansion of Fe lattice parameter with incorporation of bigger W atoms. However, the appearance of a broad shoulder at ~43° indicates that the coating contains an amorphous-like fraction. It is worth noticing that appearance of crystalline peaks in Fe-W alloy having ~ 20 at.% of W is rather unusual because at the W contents above ~16 at.% the transformation to amorphous structure typically occurs (Nicolenco et al., 2018a). In fact, the deposition of Fe-W alloys previously reported by different authors (Bobanova et al., 2009; Wang et al., 2016) was performed under stagnant conditions, while the effect of the hydrodynamic conditions on the structure development during electrodeposition of this type of alloy has not been investigated yet. It was, however, observed that under stirring conditions the adsorption state of an intermediate electroactive complex and the pH of the near-electrode layer are modified as compared to stagnant conditions, thus leading to the growth of coatings with coarser grains (i.e., having stronger X-ray reflection) (Belevskii et al., 2010).

By increasing the content of alumina particles, a gradual variation in the microstructure is observed. The intensity of the Fe(W) crystalline peaks is reduced with the incorporation of a small amount of Al_2_O_3_ particles and only one broad peak is observed with further increase in concentration of alumina in solution and in the coating (Figures 3C,D). Typically, the broadening of XRD peaks indicates the decrease in grain size that is commonly observed with the incorporation of second phase particles. The particles can restrain grain growth in two ways: (i) disordering the regular structure providing more active sites for crystal nucleation and hence, by themselves, limiting the crystallites growth (Yilmaz et al., 2014; Bhogal et al., 2015); (ii) causing a shift of overpotential, thus leading to compositional variations in metallic matrix, which in turn causes a decrease (or increase) in the average grain size (Beltowska-Lehman et al., 2012; Yari and Dehghanian, 2013), that is the typical case of W alloys with iron group metals (Tsyntsaru et al., 2012). As has been discussed above, the incorporation of alumina particles does not change significantly the W content in Fe-W matrix. Therefore, it is possible to separate the direct contribution of reinforcement particles to the grain refinement of the composite from the amorphization of the electrodeposited matrix due to the W content variation.

**Figure 4:**
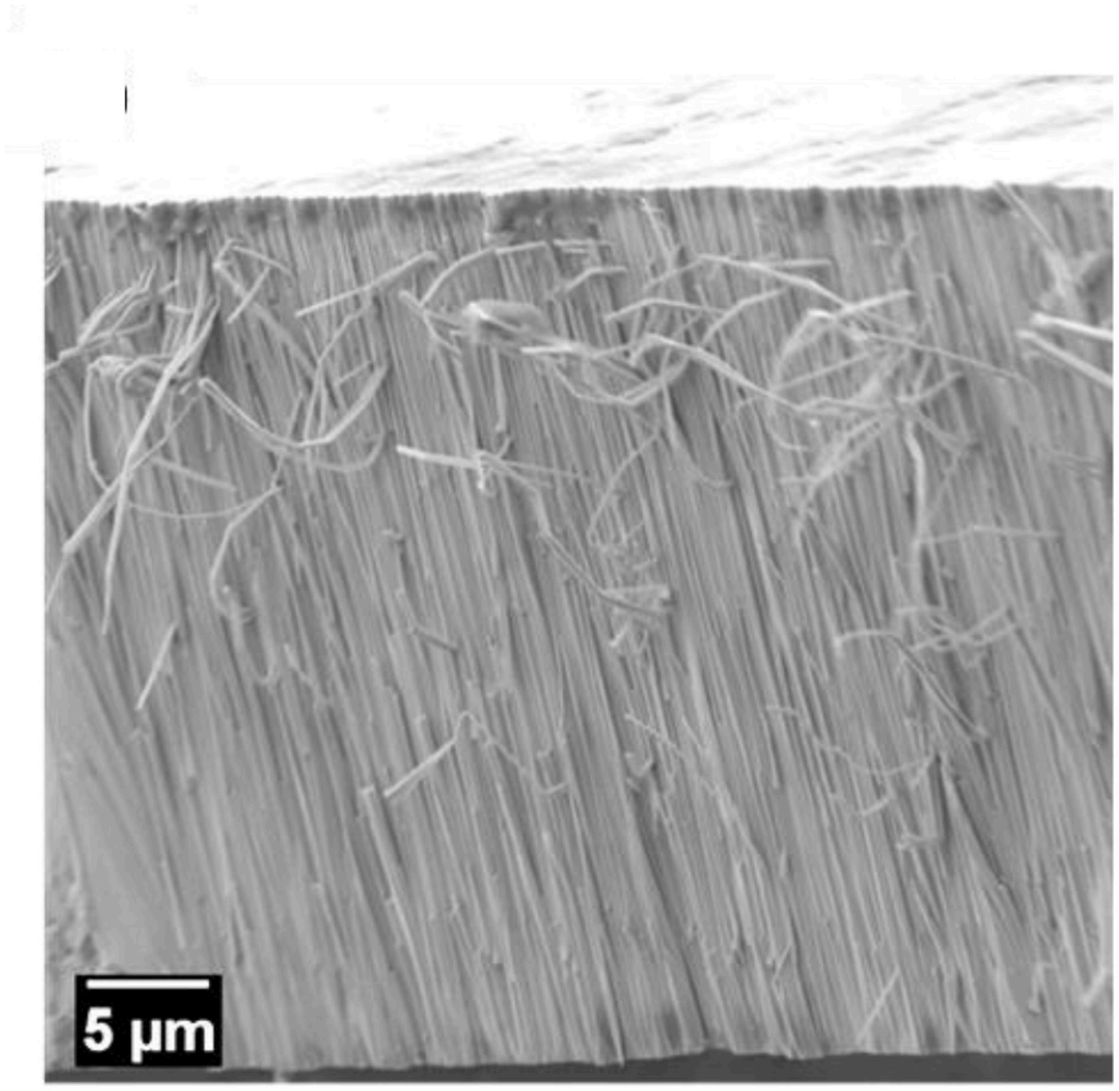
SEM micrograph of a fracture surface of the Fe-W coating showing a fibrous structure.

To get further insight into the structure of the coatings, EBSD analyses were performed on selected areas of the coatings cross-sections. During metallographic preparation of the samples the formation of columns growing perpendicular to the substrate plane was noted in the fracture surface of the Fe-W alloys (Figure 4). A previous study suggested the formation of nanofibers in Ni-W and Co-W alloys rich in W electrodeposited onto copper substrate (Donten et al., 2003), while another study on Co-W alloys revealed a columnar structure only for the coatings with low W content, i.e., around 3 at.% (Tsyntsaru et al., 2013). This implies that the columnar structure is strictly determined by the electrochemical and hydrodynamic conditions, whose modification may greatly change the nucleation and growth processes. As a matter of fact, no columnar structure was revealed in Fe-W alloys electrodeposited under stagnant conditions (Nicolenco et al., 2018a).

**Figure 5:**
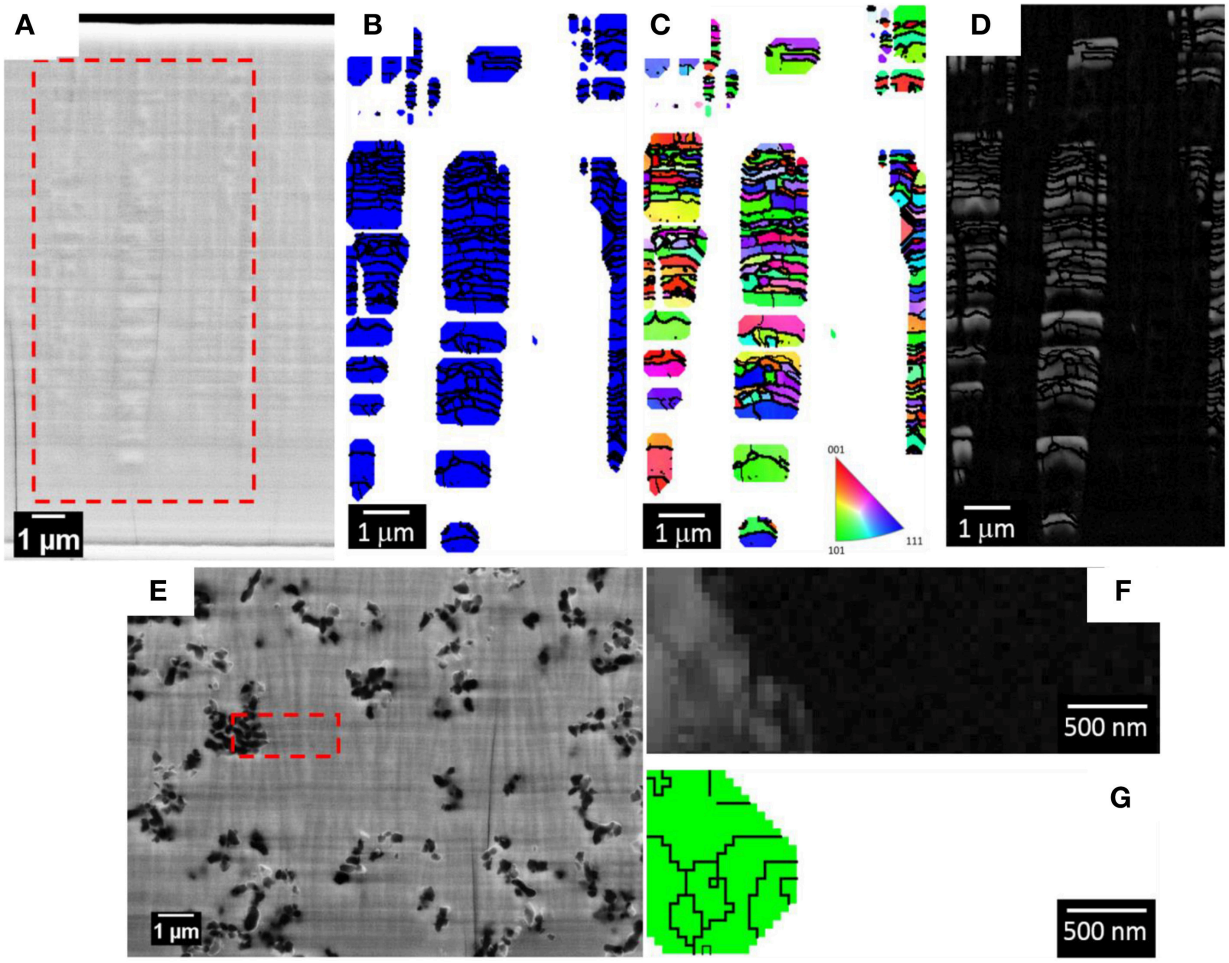
SEM micrograph of the polished cross-section of the Fe-W coating for EBSD analysis **(A)**. The red dashed box is highlighting the area from where the EBSD maps were acquired: phase map **(B)**, orientation map in inverse pole figure coloring along growth direction with the corresponding code **(C)**, and band contrast map **(D)**. In the phase map the blue grains belong to the Fe(W) phase. SEM micrograph of the polished cross-section of the sample deposited with 100 g L^−1^ of Al_2_O_3_ particles for EBSD analysis **(E)** with a band contrast map **(F)** and phase map **(G)**. In the phase map the green grains belong to the Al_2_O_3_ phase. The zero solutions are indicated as white pixel in both the orientation and phase maps, as dark pixel in the band contrast map.

Figure 5A shows a secondary electron image of the polished cross-sections of the sample deposited with 0 g L^−1^ of Al_2_O_3_ particles (Fe-W alloy) and the red dashed box defines the area where the EBSD phase map (Figure 5B), orientation map (Figure 5C), and band contrast map (Figure 5D) were acquired. A Fe(W) solid solution phase (i.e., bcc cubic cell with 2.9231 Å lattice constant), generated from the XRD results (Figure 3B) was used as the reference phase for the EBSD indexing. As shown in the SEM image in Figure 5A, the 50 nm polishing of the cross-section reveals the presence of pillars of various thicknesses. EBSD analysis performed at these areas shows that such pillars consist of several sub-micron/nano Fe(W) grains, presented in blue in the phase map of Figure 5B. The majority of Fe(W) grains have a diameter between 50 and 250 nm. The Fe(W) grains are characterized by random texture, as there is no predominant color in the orientation map shown in Figure 5C. A large fraction of zero solutions, that is 71%, is found in between the pillars. Zero solutions are shown in the phase and orientation maps as white pixels, while in the band contrast map as dark pixels. The band contrast map shown in Figure 5D is providing information about the quality of the EBSD patterns acquired from the analyzed area: crystalline regions appear bright, while areas providing poor quality patterns (e.g., grain boundaries, amorphous phase, or deformed grains) appear dark. Hence, the absence of EBSD pattern in between the Fe(W) pillars could be related to an amorphous phase, or to a nanocrystalline phase with sizes below the EBSD detection limits, that is 20 nm. Such high fraction of zero solutions would be expected considering the broad shoulder in XRD pattern of the sample, as can be seen in Figure 3B.

Figure 5E shows a secondary electron image of the polished cross-sections of the sample deposited with 100 g L^−1^ of Al_2_O_3_ particles and the red dashed box defines the area where the EBSD band contrast map (Figure 5E) and phase map (Figure 5G) were acquired. As revealed from the SEM image of the polished cross-section, a columnar structure is found also in the Fe-W/Al_2_O_3_ composite sample. The size of the columns, both in thickness and length, appears smaller as compared to the columns found for the sample deposited with 0 g L^−1^ of Al_2_O_3_ (see Figures 5A,D). Their extension along the cross-section appears to be limited by the presence of the alumina particles. However, as shown in the band contrast map (Figure 5F), it was not possible to acquire any EBSD pattern from the areas in the cross-section that were including the columnar structures. Only areas containing Al_2_O_3_ particles were correctly indexed (the grains of the Al_2_O_3_ particles are shown with a green color in the phase map in Figure 5G). These results are in agreement with the XRD findings (Figure 3E) regarding the presence of an amorphous phase, or of a nanocrystalline phase with smaller grain sizes compared to the Fe-W alloy. Hence, as observed from XRD and EBSD results, the incorporation of Al_2_O_3_ particles in the coatings leads to a reduction of the grain size but it does not prevent the development of columnar growth. Based on these observations one can infer the inherent porosity between the columnar Fe-W matrix and incorporated spherical shape Al_2_O_3_ particles, which is however challenging to confirm experimentally.

### Effect of Alumina Particles on Mechanical Properties of Fe-W Composite Coatings

**Figure 6:**
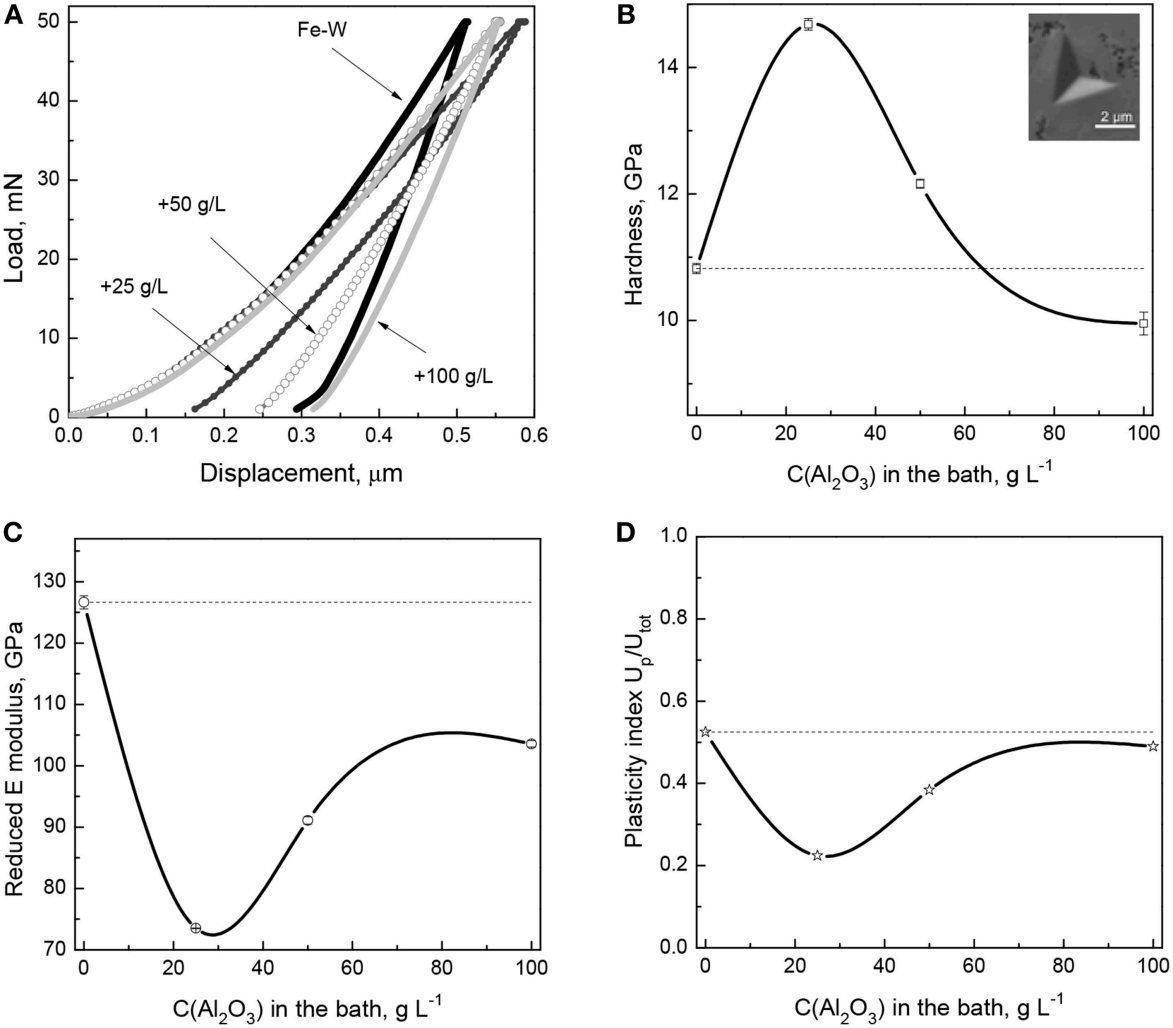
Load-displacement curves **(A)** and extracted dependence of hardness **(B)**, elastic modulus **(C)**, and plasticity index **(D)** as a function of the particles concentration in the bath. In insert **(B)** is the representative SEM image of the indentation imprint on the cross-section area of Fe-W/Al_2_O_3_ composite with lowest particles concentration.

The mechanical properties (hardness, reduced elastic modulus, plasticity index, and elastic strain to failure) of the Fe-W and Fe-W/Al_2_O_3_ coatings were obtained by nanoindentation on cross-section and the representative load–displacement curves are shown in Figure 6A. It can be seen that compared to the Fe-W matrix, the composite coatings show rather complex behavior.

The hardness values extracted from the load-displacement curves show that with the incorporation of small amounts of particles into the Fe-W matrix the hardness increases sharply from ~10 GPa to ~15 GPa (Figure 6B). It is commonly observed that the addition of particles improves the hardness of the coatings due to the two synergistic effects: (i) dispersion-strengthening, related to the presence of mechanically hard fine particles retarding the plastic deformation of the matrix, and (ii) grain refinement, resulting in an increased number of grain boundaries able to stop the dislocation motions. The former mechanism is described by the Orowan's equation, which assumes that the dislocations bend out when passing the obstacles (particles), thus creating some resisting force which leads to improved hardness of the material. However, as the concentration of incorporated particles further increases, then a continuous decrease in hardness is observed (Figure 6B), so the hardness of the composite coating with the highest particles concentration is even lower than that of pure Fe-W matrix. This can be attributed to the apparent grain size refinement/amorphization confirmed by XRD and EBSD analyses (Figures 3, 5). The mechanical behavior of a composite is then governed by the inverse Hall-Petch relation, which describes the decrease of hardness as being due to grain boundary sliding and triple junctions (i.e., plastic deformation mechanisms different than dislocation motion), similar as it was observed elsewhere (Giallonardo et al., 2011; Wasekar et al., 2016).

Contrary to what is observed for the hardness, the trend in elastic modulus is more unexpected. Figure 6C reveals that E_r_ reduces with the incorporation of a small amount of particles (25 g L^−1^ Al_2_O_3_ particles in the bath). This is in contradiction with a large number of studies showing the increase in elastic modulus with the addition of particles (Beltowska-Lehman et al., 2012; García-Lecina et al., 2013; Mahidashti et al., 2018). Often, the so-called rule of mixtures is used to infer the strengthening of composite materials containing reinforcing fibers (i.e., with columnar microstructure), which can roughly predict the elasticity of the material if the load is applied parallel to the fibers direction (isostrain conditions). Taking into account the columnar structure of Fe-W revealed in Figures 4, 5 and the rather spherical (non-columnar) shape of the reinforcing particles, it can be expected that applying the load on cross-section, i.e., perpendicular to the Fe-W fibrous, the reinforcing effect of the particles would be less pronounced than in isostrain conditions. Nonetheless, even if the microstructure of Fe-W/Al_2_O_3_ composite does not correspond to the ideal “isostrain” conditions, an increase of the Young's modulus of the composite with respect to that of the matrix should be expected, since the Al_2_O_3_ particles possess a Young's modulus larger than the Fe-W matrix. However, other factors like loose Al_2_O_3_ particles-matrix interfaces (Zhou et al., 2016) and, more in particular, nanoporosity surrounding the embedded particles could explain the reduction of E_r_. Actually, a strong dependence of the Young's modulus on porosity in nanoporous materials is well-known and it has been confirmed by nanoindentation and finite element simulations in a variety of works (Ramakrishnan and Arunachalam, 1993; Gibson and Ashby, 1999; Pellicer et al., 2012). Higher porosity causes a decrease of the reduced Young's modulus during nanoindentation (Esqué-de los Ojos et al., 2016). When the amount of incorporated particles increases, since alumina has a very high Young's modulus (300–400 GPa) the net effect is that E_r_ increases but remains always lower than for the pure Fe-W alloy.

The plasticity index was also derived from the nanoindentation data in order to provide further insight into mechanical properties of electrodeposited composite coatings. Thus, the U_p_/U_tot_ reduces with the incorporation of small amount of particles (Figure 6D), indicating rather elastic nature of the composites and their improved resistance to permanent deformation in contrast to rather plastic nature of Fe-W matrix. An increase in plasticity observed in the composites with higher particles concentration is most likely due to the grain refinement which promotes branching of the shear bands in nanocrystalline/amorphous matrix (Figure 6D).

### Effect of Alumina Particles on Tribological Properties of Fe-W Composite Coatings

**Figure 7:**
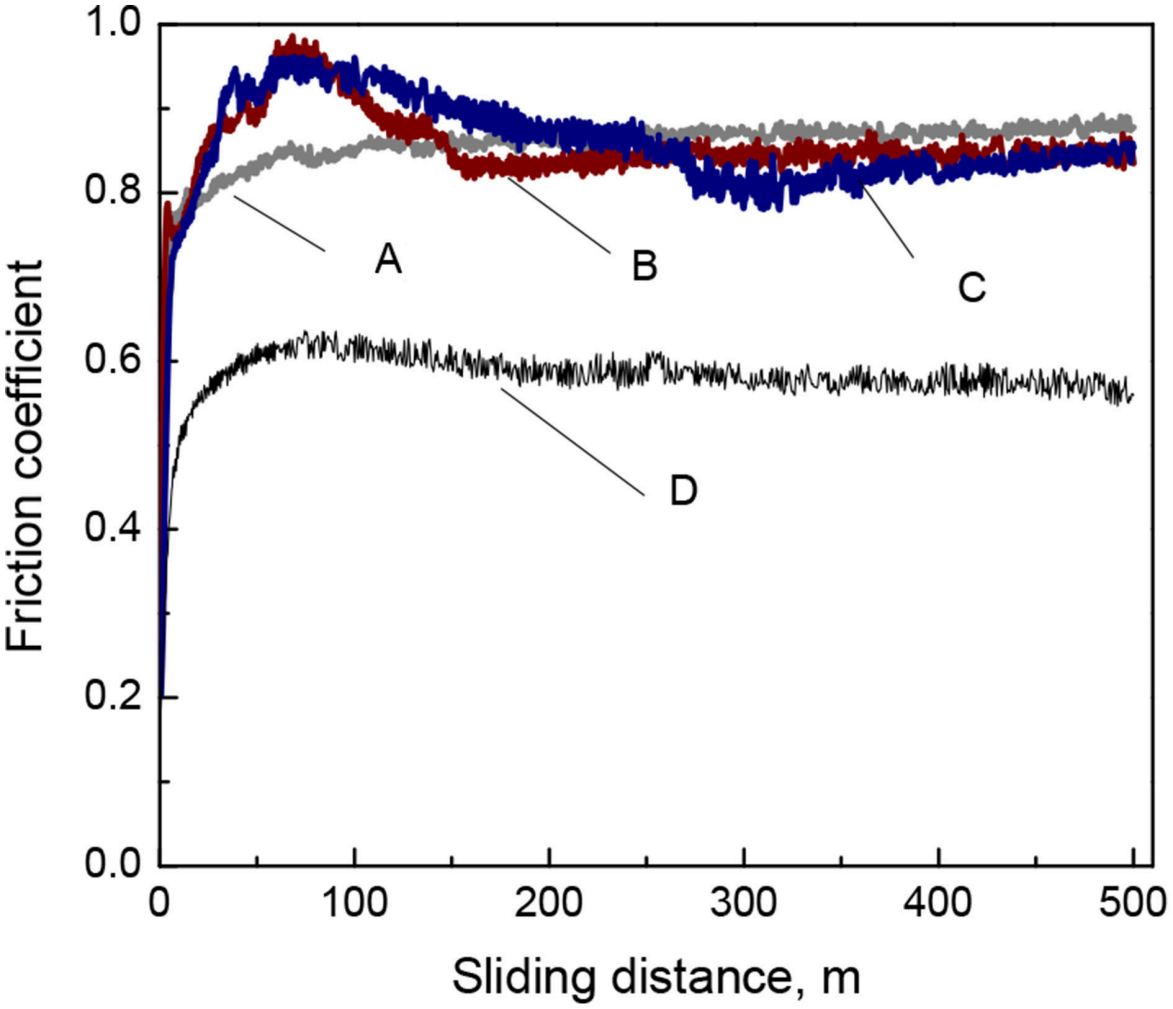
Evolution of the coefficient of friction of Fe-W **(A)** and Fe-W/Al_2_O_3_ composite coatings obtained with different concentration of alumina in electrolyte: 25 g L^−1^ **(B)**, 50 g L^−1^ **(C)**, and 100 g L^−1^ **(D)** tested under dry friction conditions at 2 N load, 4 cm s^−1^, 500 m.

The investigation of tribological behavior of electrodeposited Fe-W and Fe-W/Al_2_O_3_ composite coatings was carried out using ball-on-disk configuration sliding tests where conditions were the same for all the tested tribo-pairs. The variation of the coefficient of friction (COF) recorded during the tests as a function of sliding distance is shown in Figure 7. The COF of the Fe-W alloys and composite coatings with low particles concentration is high, ~0.8–0.9, while it decreases to ~0.6 for the composite coating with the highest particles concentration. Typically, the high values of COF are ascribed to the presence of microscopic irregularities of materials surfaces. Taking into account that the initial surface roughness of both Fe-W coatings and alumina counter body was in order of hundred nanometers, such high values of the COF can be attributed to the presence of adhered debris which increase the asperity contact between two bodies in contact.

**Figure 8:**
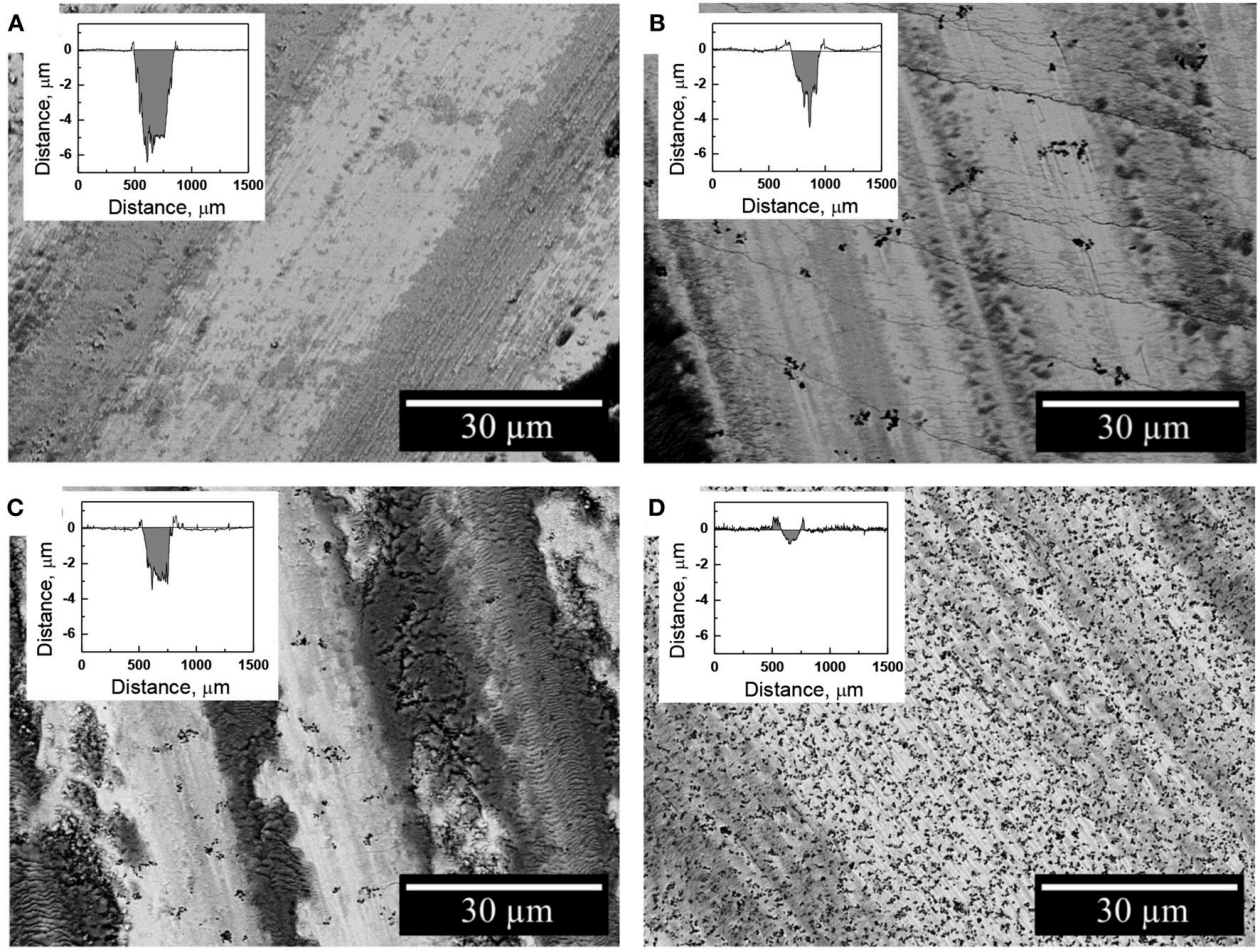
SEM images of the worn surface of the Fe-W **(A)** and Fe-W/Al_2_O_3_ coatings obtained with different concentration of alumina in electrolyte: 25 g L^−1^ **(B)**, 50 g L^−1^ **(C)**, and 100 g L^−1^ **(D)** presented along with the corresponding wear track depth profiles.

The nature of debris particles was investigated by SEM/EDS techniques on the resulted wear tracks (Figure 8). Indeed, the debris particles were found to be distributed within the whole length of the wear track accumulating mainly in piles. The oxygen content in these regions is increased up to 50 at.% which corresponds to the oxygen content in mixed iron oxide Fe_3_O_4_. This is consistent with the previous studies on the tribological behavior of electrodeposited Fe-W alloys which showed that these coatings undergo severe oxidation during fretting (tribo-oxidation) (Bobanova et al., 2009; Nicolenco et al., 2018b). The tribo-oxidation phenomenon can be explained as follows: when two surfaces slide together, the friction work is turned into thermal energy, which tends to maximize the potential energy at the interface. Since the maximized thermal energy is naturally an unstable state, the oxidation itself is a form of intrinsic response of the material to recover the system equilibrium under friction conditions (Abdel-Aal, 2003). Thus, the formation of iron oxides during dry friction can be considered a driving factor determining the resultant tribological behavior of the Fe-W coatings (COF, wear depth/volume, cracks initiation, plastic deformations, etc.). As it can be seen from Figure 8C, the presence of micro-sized Al_2_O_3_ particles significantly reduce the area of the exposed Fe-W matrix, thus reducing the asperity contact between two sliding surfaces what results in a lower COF and wear depths (Figure 8).

**Figure 9:**
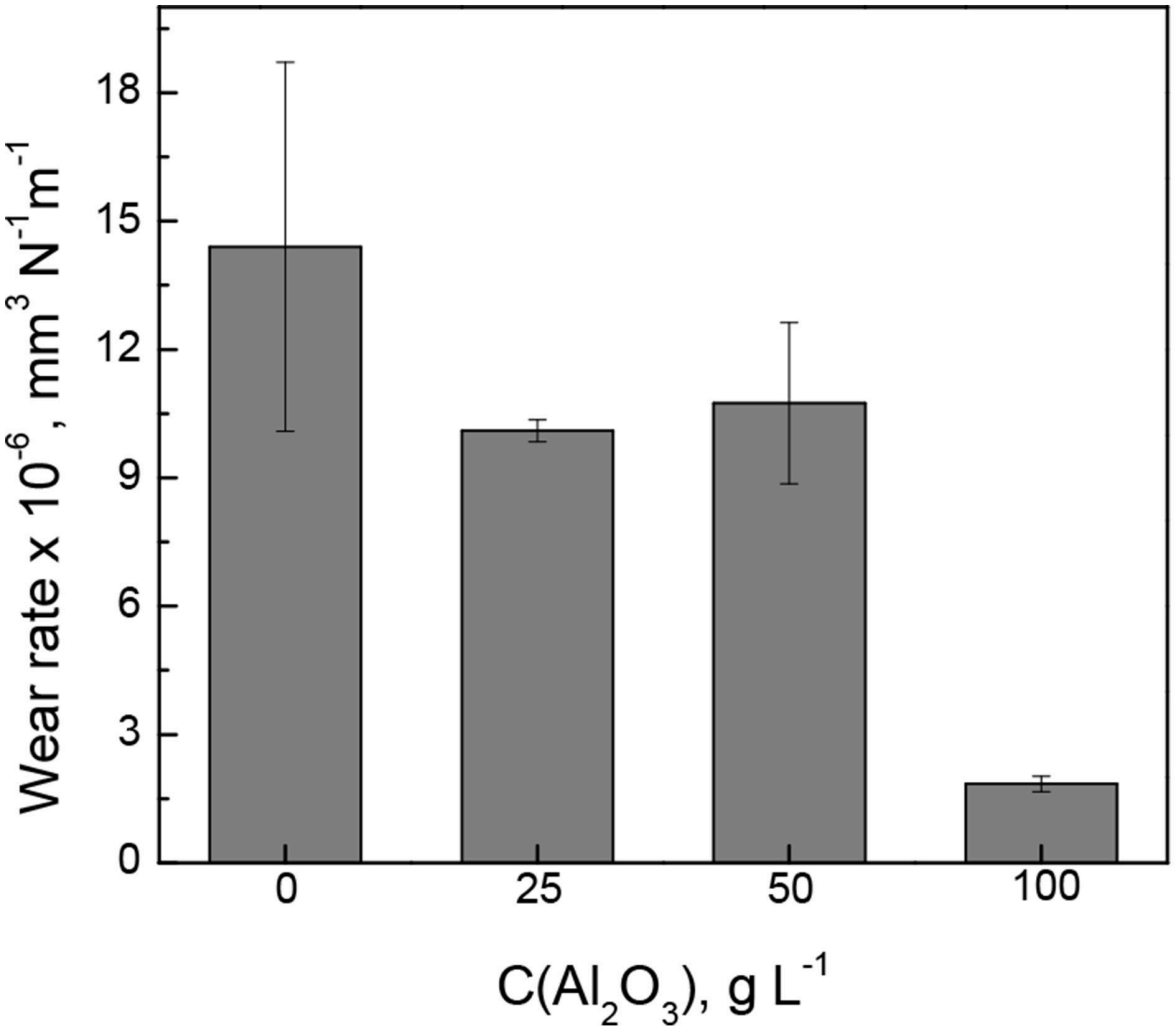
The wear rate of Fe-W alloy and Fe-W/Al_2_O_3_ composite coatings at dry friction conditions at 2 N load, 4 cm s^−1^, 500 m.

The wear rate of Fe-W alloy and Fe-W/Al_2_O_3_ composite coatings was calculated by integration the cross-section area of the wear tracks shown in inserts Figure 8 and the obtained results are shown in Figure 9. The interpretation of the depth profiles was sometimes not straightforward due to the presence of debris which remained adhered in the wear track even after ultrasound cleaning that increased the error in calculation of the wear depth and wear rate. Nevertheless, it is clearly seen that the addition of Al_2_O_3_ particles reduces the specific wear rate, i.e., increases the wear resistance of the coatings under the tested conditions. The lowest wear rate (1.8·10^−6^ mm^3^ N^−1^ m^−1^) was obtained for the sample with the highest particles concentration, which is an order of magnitude lower than that of pure Fe-W alloy matrix. It is also comparable to the wear rate of annealed Fe-W alloy and even electrodeposited hard chromium coatings tested by using a similar set-up and dry friction conditions applied (Mulone et al., 2019). Thus, in annealed Fe-W alloys the reduction in the tribooxidation was achieved mainly due to the phase transformation, that is, the formation of Fe_2_W and FeWO_4_ hard phases which are not prone to oxidation. Hence, electrodeposited Fe-W/Al_2_O_3_ composite coatings are getting advantageous in terms of fabrication (one-step electrodeposition, no post-treatment required, environmentally-friendly bath composition) and sustainability (suitable for chromium replacement). Taking into account the outstanding thermal resistance of the amorphous-like Fe-W deposits (Mulone et al., 2018a) (up to 600°C) and remarkable hardness of these coatings (10–16 GPa), the novel Fe-W/Al_2_O_3_ composites can be considered as alternative appealing materials for a wide range of technically-demanding applications, such as components and mechanisms where lubrication is recognized as ineffective or inapplicable, e.g., in high-temperature vacuum systems.

Many researchers address the importance of the mechanical characteristics for the good wear resistance and show a linear relationship between the hardness (or H/E ratio) and wear for different polycrystalline materials like Ni-W (Sriraman et al., 2006), Ni-P (Jeong et al., 2003), and different type of composites (Leyland and Matthews, 2000; Shi et al., 2006; García-Lecina et al., 2013; Man et al., 2014; Bajwa et al., 2016). It is assumed in these cases that the abrasive wear originates from plastic deformation. Therefore, materials with higher H (or H/E ratio) should better resist the plastic deformations and thus have lower wear. However, in the Fe-W system the tribological behavior is primarily governed by the tribo-oxidation (Mulone et al., 2019), i.e., the chemical reactivity of the material and the nature of the oxidation products formed are the determining factors. Nevertheless, the high hardness of Fe-W/Al_2_O_3_ composites may result in an excessive coating brittleness contributing to the cracks propagation inside the wear tracks as depicted in Figures 8B,C.

### Effect of Alumina Particles on Corrosion Properties of Fe-W Composite Coatings

**Figure 10:**
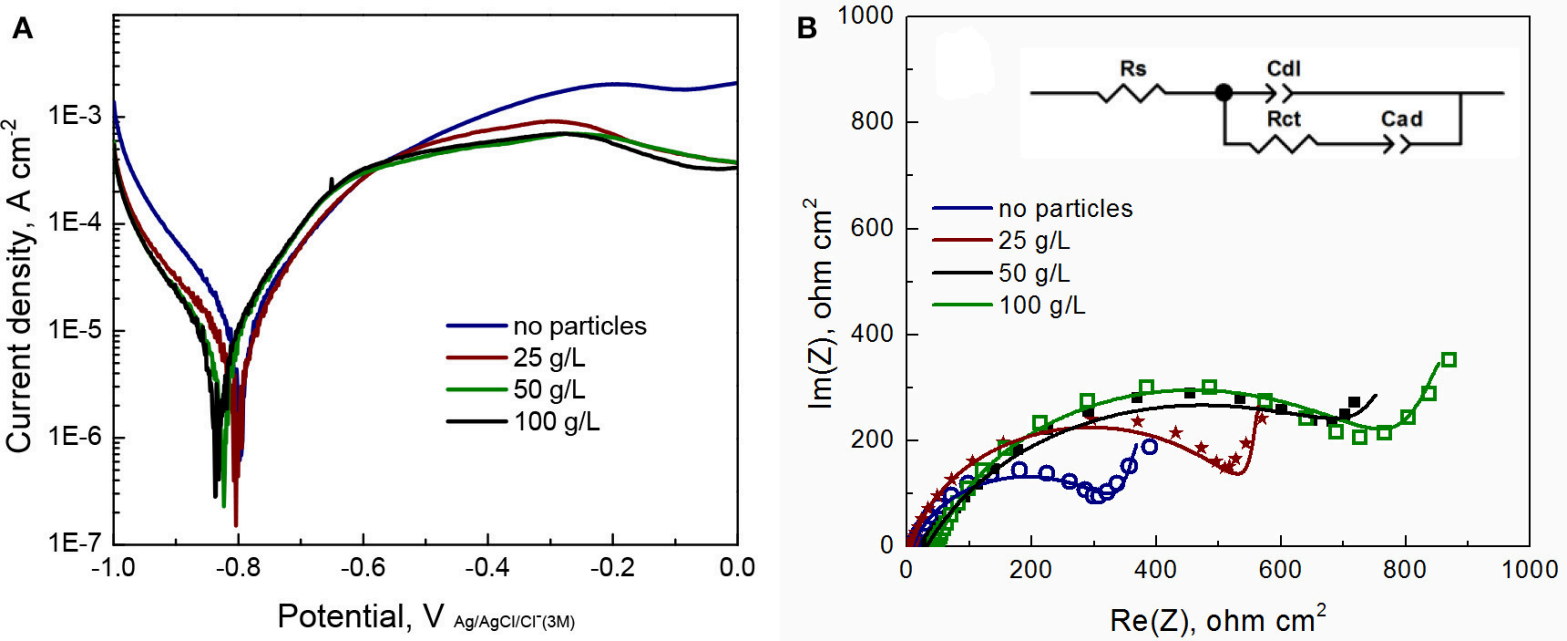
Linear sweep voltammograms **(A)** and EIS Nyquist plots **(B)** for Fe-W and Fe-W/Al_2_O_3_ coatings electrodeposited under similar conditions and examined in 0.1 M NaCl solution at OCP. In insert **(B)** is the electrochemical circuit used for data fitting. R_s_, solution resistance; R_ct_, charge transfer resistance; C_dl_ is a constant phase element having meaning of the double layer capacitance of alloy surface and C_ad_ is adsorption-related capacitance of intermediates.

Corrosion of Fe-W alloys and composites was induced by using 0.1 M NaCl solution, which is commonly used to simulate marine environment. The obtained potentiodynamic polarization curves and Nyquist plots are presented in Figures 10A,B, respectively, and the extracted parameters are given in the Table 1. It can be seen from Figure 10A that the Fe-W based coatings exhibit a very similar behavior with an active anodic metal dissolution that do not transfer into passive state. With the increase in particles concentration the corrosion potential (E_corr_) slightly shifts toward more negative potentials. Usually, the lowering of corrosion potential indicates a higher tendency of the electrode to be dissolved in an electrolyte. Nevertheless, different hydrogen overvoltage on Fe-W surface with different alumina contents may vary without a direct correlation with the corrosion rate. Moreover, accumulation of corrosion products on the surface could also cause the lower values of E_corr_. The corrosion current density (j_corr_) was calculated from the presented polarization curves. Usually, the corrosion current is determined by the extrapolation of the rectilinear dependences of log j vs. E to E_corr_ in the Tafel region. However, due to the peculiarities of cathodic hydrogen evolution and active anodic metal dissolution the cathodic and anodic branches of recorded voltammograms are asymmetric (Figure 10A), what makes the standard j_corr_ determination not reliably applicable. Therefore, the values of j_corr_ were obtained by using Allen-Hickling equation which enables to accurately estimate the corrosion current from the data obtained in a relatively narrow potential range close to E_corr_ (Kublanovsky et al., 2008; Vernickaite et al., 2016). The obtained results show that the corrosion current density for all the studied samples is the same order, independently of the content of co-deposited alumina particles (Table 1).

**Table 1:** Extracted corrosion parameters from E vs. j plots shown in Figure 10A and corresponding equivalent circuit parameters determined by fitting the impedance spectra of electrodeposited Fe-W and Fe-W/Al_2_O_3_ coatings (Figure 10B) in 0.1 M NaCl medium at room temperature.

**Coating**	**–E_**corr**_, V**	**–j_**corr**_, A/cm^**2**^**	**R_**ct**_, Ω cm^**2**^**	**C_**dl**_, F/ cm^**2**^**	**n(C_**dl**_)**	**C_**ad**_, F/ cm^**2**^**	**n(C_**d**_)**	**R_**s**_, Ω cm^**2**^**
Fe-W	0.795	5 × 10^−4^	355.2	1.54 × 10^−3^	0.80	6.36 × 10^−2^	0.89	8.8
Fe-W+25 g/L Al_2_O_3_	0.804	3 × 10^−4^	569.1	8.61 × 10^−4^	0.85	5.66 × 10^−2^	0.95	6.9
Fe-W+50 g/L Al_2_O_3_	0.823	3 × 10^−4^	843.8	1.55 × 10^−3^	0.71	6.92 × 10^−2^	0.98	31.2
Fe-W+100 g/L Al_2_O_3_	0.837	3 × 10^−4^	795.1	8.18 × 10^−4^	0.81	3.32 × 10^−2^	0.85	45.6

“*n”—is the exponent in the equation for the constant phase element that measures how far the interface is from an ideal capacitor (0 < n < 1)*.

In the Figure 10B, each point in the plot corresponds to the measured impedance at a certain frequency, while the continuous line presents the best-fit data obtained by using an equivalent circuit shown in insert Figure 10B. The used equivalent circuit for fitting of EIS data supposes the complicated mechanism of alloy corrosion, which perhaps involves intermediate stages with the adsorption of corrosion products (Vernickaite et al., 2016). The equivalent circuit used to simulate the EIS consisted of R_s_–solution resistance (uncompensated resistance); R_ct_–charge transfer resistance; C_dl_–a constant phase element having meaning of double layer capacitance of the electrolyte at alloy surface; and Cd which is adsorption-related capacitance of intermediates. Accordingly, circuit R_ct_C_dl_ describes the Faraday process, i.e., the resistance and capacities of the layer against electrochemical reaction, and Cad represents the process of blocking the coating's surface by adsorbed species. Generally, the higher the charge transfer resistance R_ct_, the greater corrosion resistance of the system. Thus, according to Table 1, the lowest corrosion resistance was obtained for the Fe-W coating without particles. The highest values R_ct_ are obtained for the composite coatings produced from the bath with 50 and 100 g L^−1^ of particles (6 and 12 vol% Al_2_O_3_, respectively).

Noticeably, the R_ct_ obtained for Fe-W coatings electrodeposited from glycolate-citrate electrolyte and tested in chloride medium can be roughly comparable to other Fe-containing deposits described in literature: Fe-W alloy electrodeposited from citrate bath and tested in sulfate-chloride medium (380.7 Ω cm^2^) (Vernickaite et al., 2015), Ni-Fe-W alloy tested in similar corrosion medium (207.0 Ω cm^2^) (Sriraman et al., 2007) and with Ni-Fe/Al_2_O_3_ composite in sodium sulfate (~250 Ω cm^2^) (Starosta and Zielinski, 2004); whereas the corrosion resistance of Co-W and Ni-W alloys in neutral mediums is much higher, i.e., 1,200 and 6,770 Ω cm^2^, respectively (Sriraman et al., 2007; Vernickaite et al., 2016). Taking this into account, and the small variations in corrosion currents obtained for investigated Fe-W composite coatings, one could suspect the detrimental and prevalent effect of Fe dissolution from the coating.

**Figure 11:**
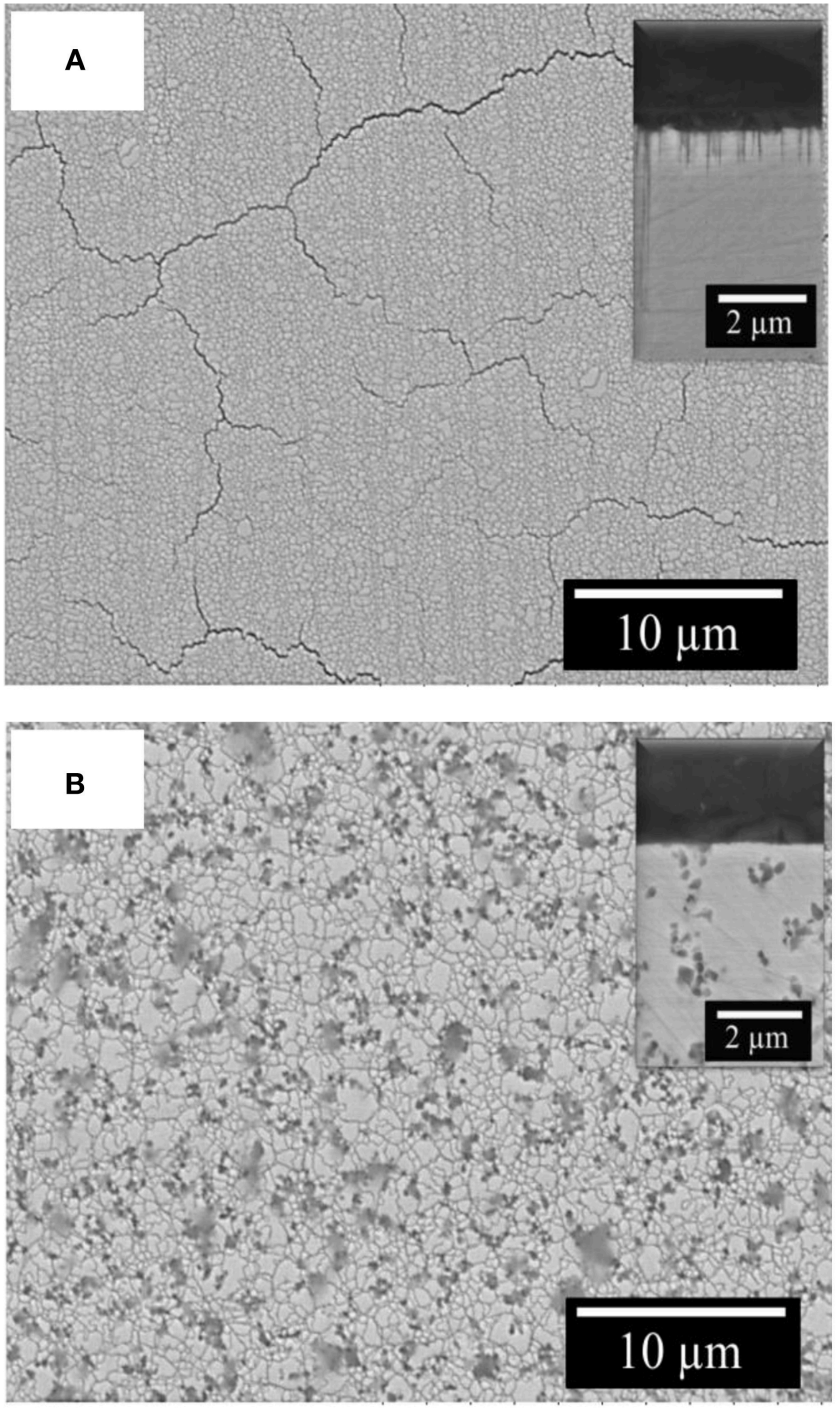
SEM images of Fe-W **(A)** and Fe-W+100 g/L Al_2_O_3_ coatings **(B)** after corrosion test in 0.1 M NaCl at room temperature. In inserts are the corresponding cross-sectional images of the samples.

The surface of investigated Fe-W coatings after corrosion test is cracked and the cracks propagation is along the grain boundaries as it can be observed in Figure 11A. The corrosion medium can penetrate faster through these cracks, thus accelerating the dissolution of Fe-W matrix. The EDS analysis of corroded surface of the studied coatings showed a significant increase in both oxygen content (up to 50 at.%) and the W content (up to 28 at.%), which is in accordance with other studies performed on W alloys with Fe group metals (Wang et al., 2014; He Y. et al., 2016; Vernickaite et al., 2016). This indicates that the corrosion process occurs through the preferential dissolution of Fe from the matrix, which leads to the formation of iron oxides and hydroxides at the electrode surface and to release of free Fe^2+^ ions into solution (Sriraman et al., 2007; He Y. et al., 2016). It is supposed that during corrosion process W preferentially form oxides, or can be also released into solution in form of ${\text{WO}}_{4}^{2-}$ ion. Thus, FeWO_4_ and Fe_2_(WO_4_)_3_ compounds could be found in corrosion products, as shown in He et al. (2016). Slightly increased corrosion resistance shown by EIS can be attributed to the presence of alumina particles which partially block the surface and hinder the propagation of corrosion cracks, thus no cracks are evidenced on the surface and cross-section of the composite coatings after corrosion test (Figure 11B).

## Conclusions

The electrodeposition of novel Fe-W/Al_2_O_3_ composite coatings with various alumina concentrations has been carried out from an Fe(III)-based electrolyte. The addition of alumina particles with concentrations ranging from 25 to 100 g L^−1^ to the Fe-W plating bath did not influence the W content in Fe-W matrix and the deposition rate remains also virtually unchanged. The content of co-deposited Al_2_O_3_ increased linearly from 2 to 12 vol% with the increase in particles concentration in the bath. The obtained Fe-W and Fe-W/Al_2_O_3_ composite coatings were characterized by a columnar growth structure. In the Fe-W coating such columns consisted of several sub-microns and nano Fe(W) grains. As observed from XRD and EBSD results, the incorporation of alumina particles in the Fe-W/Al_2_O_3_ composite coatings led to a reduction of the grain size and amorphization, but it did not prevent the development of columnar growth. These structural changes partially contributed to an increase in measured hardness up to 15 GPa obtained with the addition of small amount of particles (25 g L^−1^ in the bath, 2 vol% in deposit), followed by a decrease below 10 GPa for the coating with maximum volume fraction of alumina. The dependence of the elastic modulus on the Al_2_O_3_ particles concentration is not the typical one expected for composite coatings with reinforcing particles. For low particles concentration the Young's modulus decreases by around 40% and it increases with further increase of the particles concentration, but still remains lower than for the pure Fe-W alloy. The observed reduction in Young's modulus could be explained by the presence of nanopores surrounding the particles, among other possible reasons. A superior tribological behavior, the lowest wear rate of 1.8·10^−6^ mm^3^ N^−1^ m^−1^, was obtained for the Fe-W/Al_2_O_3_ composite coating with the highest particles concentration (100 g L^−1^ in the bath, 12 vol% in deposit). The wear rate was reduced by an order of magnitude as compared to the pure Fe-W alloy matrix mainly due to the reduction of the exposed area of the Fe-W matrix and lowering the tribo-oxidation. Electrodeposited Fe-W and Fe-W/Al_2_O_3_ composite coating show similar corrosion behavior in 0.1 NaCl medium. The corrosion occurs via the formation of Fe and W oxygen compounds and preferential Fe dissolution from the matrix independently from alumina particles concentration. Nevertheless, the presence of Al_2_O_3_ in the deposits results in a slightly increase corrosion resistance (up to ~800 Ω cm^2^) obtained by EIS due to the partial blocking of the surface and hindering the propagation of corrosion cracks.

The results presented in this work provide a possibility to integrate the nanocrystalline Fe-W/Al_2_O_3_ composite coatings into various mechanical systems working under dry friction, and in particular those working at elevated temperatures.

## Author Contributions

The electrodeposition of the coatings, wear, and corrosion measurements were performed by AN together with NI, under the supervision of NT, HC, and EG-L. The structural characterization was performed by AM under the supervision of UK. The mechanical characterization was performed by JS and EP. Writing-original draft preparation by AN and AM. All the authors contributed to the writing-review and editing.

### Conflict of Interest Statement

The authors declare that the research was conducted in the absence of any commercial or financial relationships that could be construed as a potential conflict of interest.
