# Supplementary material for: Nanocrystalline Electrodeposited Fe-W/Al2O3 Composites: Effect of Alumina Sub-microparticles on the Mechanical, Tribological, and Corrosion Properties
Source: Front Chem. 2019 Apr 16;7:241. doi: 10.3389/fchem.2019.00241 (PMC6476963; doi:10.3389/fchem.2019.00241)
Supplement: Supplementary file 1 [file Data_Sheet_1.docx]

Nanocrystalline electrodeposited Fe-W/Al_2_O_3_ composites: effect of alumina sub-microparticles on the mechanical, tribological and corrosion properties

Aliona Nicolenco* ^1,2^, Antonio Mulone^3^, Naroa Imaz^4^, Natalia Tsyntsaru* ^1,2^, Jordi Sort^5,6^, Eva Pellicer^6^, Uta Klement^3^, Henrikas Cesiulis^1^, Eva García-Lecina^4^

^1^Vilnius University, Department of Physical Chemistry, Vilnius LT-03225, Lithuania

^2^Institute of Applied Physics, Chisinau MD – 2028, Moldova

^3^Chalmers University of Technology, Department of Industrial and Materials Science, Gothenburg SE-412 96, Sweden

^4^CIDETEC, Paseo Miramón 196, Donostia-San Sebastián E-20014, Spain

^5^Departament de Física, Universitat Autònoma de Barcelona, Bellaterra E-08193, Spain

^6^Institució Catalana de Recerca i Estudis Avançats (ICREA), Pg. Lluís Companys 23, Barcelona E-08010, Spain

*** Correspondence:**Corresponding Authors
A.N. [alionanicolenco@gmail.com](mailto:alionanicolenco@gmail.com), N.T. [ashra_nt@yahoo.com](mailto:ashra_nt@yahoo.com)

Keywords: iron alloys_1_, alumina_2_, composite coatings_3_, columnar growth_4_, wear resistance_5_.


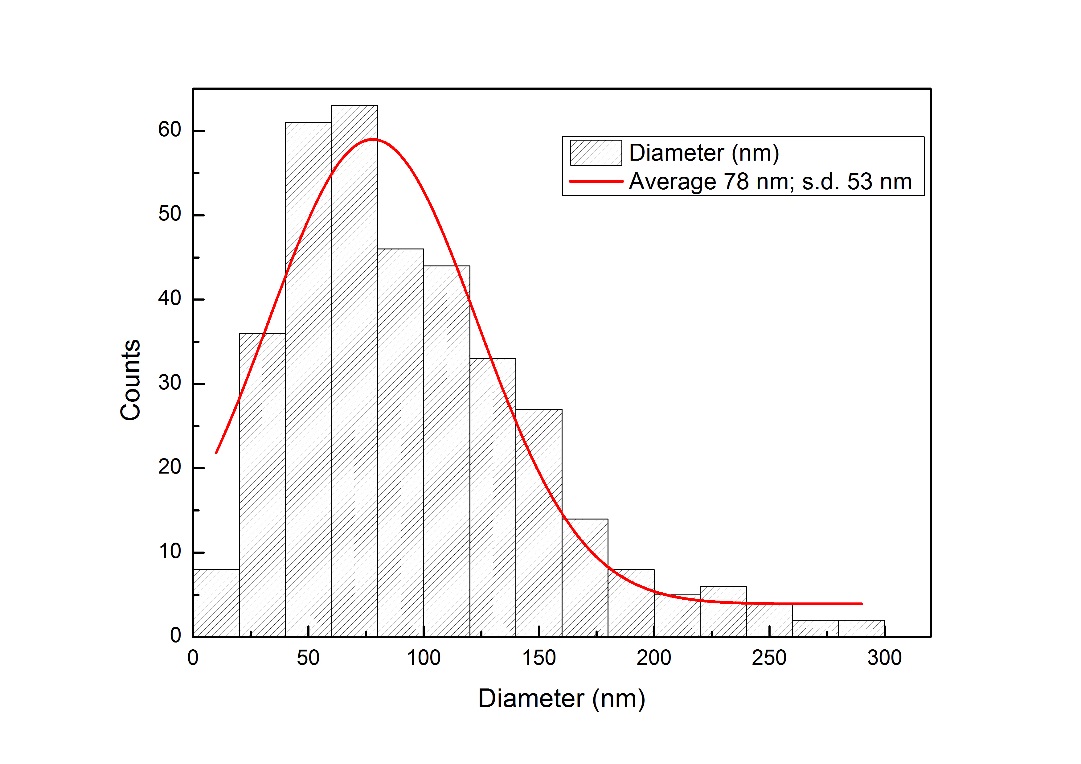


***Figure S1.*** *Alumina particles size histogram obtained by using SEM and ImageJ. Average size of Al_2_O_3_ particles is 78 nm, with a standard deviation of 53 nm.*

**
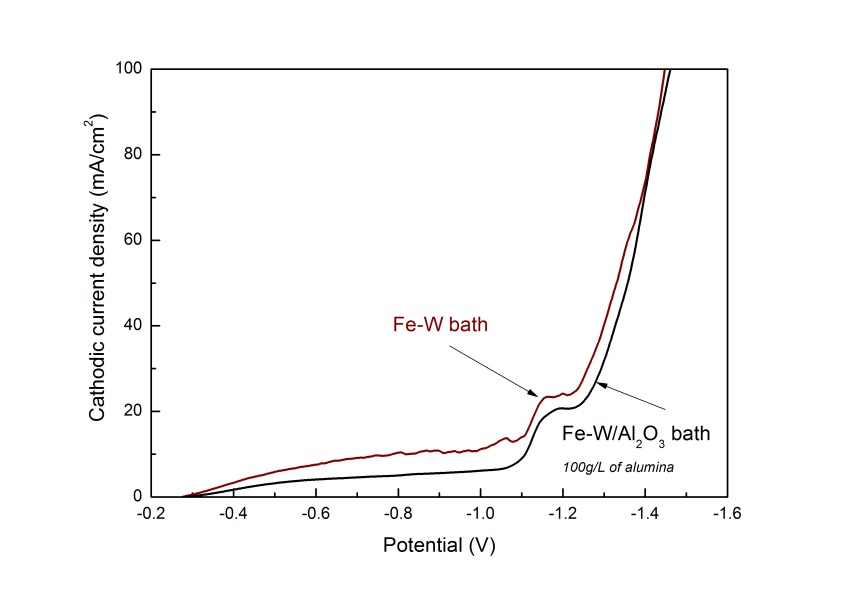
**

***Figure S2.*** *Cathodic polarization curves for Fe-W and Fe-W/Al_2_O_3_ (100 g/L Al_2_O_3_ in the bath) deposition from glycolate-citrate electrolytes obtained at 65°C and 200 rpm.*

*
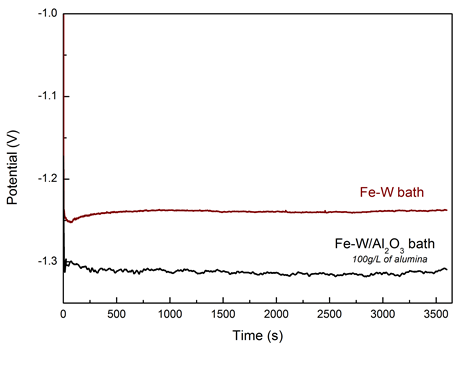
*

***Figure S3.*** *Chronopotentiometric curves for electrodeposition of Fe-W alloy and Fe-W/Al_2_O_3_ composite coatings recorded at the constant cathodic current density of 40 mA/cm^2^ at 65°C and 200 rpm.*
